# *In situ* Root Phenotypes of Cotton Seedlings Under Phosphorus Stress Revealed Through RhizoPot

**DOI:** 10.3389/fpls.2021.716691

**Published:** 2021-08-30

**Authors:** Zichen Zhang, Lingxiao Zhu, Dongxiao Li, Nan Wang, Hongchun Sun, Yongjiang Zhang, Ke Zhang, Anchang Li, Zhiying Bai, Cundong Li, Liantao Liu

**Affiliations:** State Key Laboratory of North China Crop Improvement and Regulation/Key Laboratory of Crop Growth Regulation of Hebei Province/College of Agronomy, Hebei Agricultural University, Baoding, China

**Keywords:** cotton seedlings, low phosphorus stress, root phenotypes, lateral roots, root hair longevity, rhizopot

## Abstract

Phosphorus (P) deficiency is a common challenge in crop production because of its poor mobility through the soil. The root system plays a significant role in P absorption from the soil and is the initial indicator of low P levels. However, the phenotypic dynamics and longevity of cotton roots under P stress remain unknown. In this study, RhizoPot, an improvised *in situ* root observation device, was used to monitor the dynamics of root phenotypes of cotton seedlings under P-deficient (PD) and P-replete (PR) conditions. Low P stress reduced P absorption and accumulation in the roots, leading to low dry weight accumulation. Cotton seedlings responded to low P stress by increasing the number of lateral roots, specific root length, branch density, root length density, and length of root hairs. Additionally, the life span of root hairs was prolonged. Low P stress also reduced the average diameter of roots, promoted root extension, expanded the root coverage area, and increased the range of P acquisition. Principal component analysis revealed that the net root growth rate, root length density, root dry weight, P absorption efficiency, average root hair length, and taproot daily growth significantly influenced the cotton root architecture. Collectively, these results show that low P stress reduces the net growth rate of cotton seedling roots and restricts plant growth. Plants respond to P deficiency by extending the life span of root hairs and increasing specific root length and lateral root branch density. This change in root system architecture improves the adaptability of plants to low P conditions. The findings of this study may guide the selection of cotton varieties with efficient P utilization.

## Introduction

The world population is likely to reach 9 billion people within the next 30 years, precipitating severe agricultural production challenges globally (Tyczewska et al., 2018). Phosphorus (P) is a non-renewable resource necessary for agricultural production. At present, the P absorption rate by crops is only 10–20% (Holford, 1997). Most P is fixed by minerals or organic matter in the soil and hence unavailable for plant uptake. Inefficient P utilization is one of the most important restrictive factors for global agricultural development today (Lynch, 2007; MacDonald et al., 2011). P deficiency reduces crop yield, whereas large-scale phosphate mining pollutes the environment (Withers et al., 2014). Therefore, it is necessary to explore the mechanism of P acquisition and utilization by plants in order to improve P utilization efficiency.

The root system anchors plants in the soil and is the main P absorption organ (Lynch, 2011). Plants mainly employ two strategies to increase P availability under low P stress. One involves physiological and biochemical adjustments, including the exudation of small molecular weight organic acids or extracellular acid phosphatase (Withers et al., 2014; Krishnapriya and Pandey, 2016). The other strategy involves changing the root configuration to improve P absorption (Wasaki et al., 2003; Gahoonia and Nielsen, 2004b). Root architecture determines the absorption range and distribution density of roots and, therefore, affects the amount of P that can be absorbed from the soil (Kawa et al., 2016). Studies have shown that by increasing the length of the root system, the number of lateral roots (LRN), and the branching angle of the root system, plants increase the contact area between the root system and the soil, thereby increasing P absorption (Lynch and Brown, 2001; Gahoonia and Nielsen, 2004a). Lateral roots are the most active part of the root system and thus are indicators of plant growth environment and health (Zhang et al., 2000). The lateral roots of different crops exhibit various responses to low P stress. For example, maize responds to P deficiency by increasing the lateral root branching density (LRD), shortening root length, reducing root depth, and increasing foraging on the topsoil (Jia et al., 2018; Sun et al., 2018). On the contrary, kidney bean plants respond to low P stress by reducing the number of lateral roots and LRD, while increasing the length of the root system (Borch et al., 1999; Strock et al., 2018). In *Arabidopsis* and water hyacinth, low P stress reduces the diameter of lateral roots and increases the branching density and length of lateral roots (Williamson et al., 2001; Xie and Yu, 2003; Hanlon et al., 2018). Studies on cotton show that P deficiency increases root length (Mai et al., 2018; Chen et al., 2020).

Root hairs are formed through the extension of root epidermal cells. Their primary function is to increase the contact area between the root system and the soil, thus improving the absorption efficiency of water and nutrients (Bengough et al., 2011). Both genetic and environmental factors influence the growth and development of root hairs (Datta et al., 2011; Hao et al., 2012; Giri et al., 2018). Among them, soil nutrient supply is the most significant one (Konno et al., 2006). The root hair length and density will increase in P-deficient soil, which will help to maximize P absorption (Bengough, 1997; Brown et al., 2012). For example, low P stress increased the length and density of root hairs in *Arabidopsis thaliana* (Stetter et al., 2015). Similar observations have also been reported in rice (Nestler et al., 2016). Crop varieties that are more adapted to low P stress typically have longer root hairs (Gahoonia et al., 2001). Root hairs play an important role in P absorption (Gahoonia and Nielsen, 1998); however, it is still unclear how cotton root hairs respond to low P stress. Root hairs play a significant role in P absorption in plants. Although their life span is short (2–3 weeks) (He et al., 2006), they account for up to 80% of total P absorption (Jungk, 2001). Therefore, exploring root hair phenotypes and longevity under low P stress is crucial for identifying and developing appropriately adapted crop varieties.

The root system is hidden under the soil and therefore is impossible to observe directly. As such, studying root phenotypes, especially *in situ* root system phenotypes, is a great challenge. Traditional root phenotype research methods, such as root drilling, excavation, and soil block methods, require the root system to be separated from the original growth environment. This destroys the root system structure, leading to large measurement errors. Thus, such methods cannot be used to observe the dynamic root system phenotype (Zhang et al., 2000). The current modern methods for *in situ* root phenotype research include X-ray computed tomography (XCT) (Koenig et al., 2008) and magnetic resonance imaging (MRI) (Metzner et al., 2015). However, these methods cannot be applied in a large-scale study because they are expensive, prone to error, and subject to moisture interference (Shuang et al., 2020). The *in situ* observation method of gel culture is cheap but associated with several challenges (Bengough et al., 2004), including a short culture period, limited nutrient supply, heterogeneous gel densities, and partial dehydration of the root system (Yokawa et al., 2013; Joshi et al., 2017). The emergence and development of the minirhizotron method made up for the shortcomings of the methods mentioned above (Arteca and Arteca, 2000). However, research is still required to determine how to best account for the presence of stones and coarse particles, to refine estimates of the mass density of fine roots, and to test if the growth of new roots from roots already on the tube surface creates a bias in the final productivity estimates (Bernier and Robitaille, 2004). *In vitro* methods of studying root hairs involve using a stereomicroscope to capture root hair phenotype information. These methods involve dehydration and fixation steps, which tend to change the original phenotype and cannot accurately capture dynamic information (Guo et al., 2009; Khandan-Mirkohi and Schenk, 2009). Affordability, accuracy, and efficiency are key factors to consider while developing novel methods of studying root phenotypes. In this study, the improvised *in situ* root cultivation device RhizoPot was employed to study root phenotypic characteristics. RhizoPot is assembled from acrylic plates, and a scanning plate with a scanner is placed on an inclined surface to continuously obtain high-definition *in situ* images of fine roots and root hairs. It is necessary to connect the scanner to a portable computer when collecting images (Xiao et al., 2020). The device enables *in situ*, accurate, and continuous observation of the root system without destroying the original growth conditions of the root system.

Cotton (*Gossypium hirsutum* L.) is an economically important crop worldwide and provides natural fiber, the primary raw material used in the textile industry. Low P stress is one of the main factors restricting the production of cotton. P deficiency negatively impacts cotton agronomic traits and reduces plant height, leaf area, and dry matter quality (Li et al., 2020). These changes also affect the root morphology (Chen et al., 2019). However, the dynamics of morphological changes of cotton roots under P-deficient conditions, especially the response of lateral roots and root hairs to P-deficient stress, are still unclear. In this study, root phenotypic characteristics of two cotton varieties were examined *in situ* using a root culture device improvised in our laboratory (Xiao et al., 2020). This device achieved higher efficiency, lower cost, easy operation, and high-resolution imaging and can continuously obtain phenotypic images of cotton roots non-destructively under P-deficiency stress. This study aimed to evaluate the morphological changes of cotton roots under low P stress and examine the response characteristics of lateral root phenotype and root hair longevity to low P stress. The findings of this study provide new insights into the phenotypic characteristics of cotton roots under P stress and may be applied to guide the selection of new cotton varieties with high P utilization efficiency.

## Materials and Methods

### Plant Material and Experimental Design

The experiment was conducted in 2020 at the Hebei Agricultural University (38°85′N, 115°30′E), Hebei Province, China. For the accuracy of the results, two local commercial cotton (*G. hirsutum* L.) cultivars, “Nongdamian No. 10” (ND) and “Jimian 315” (JM), were used in this study.

**Figure 1:**
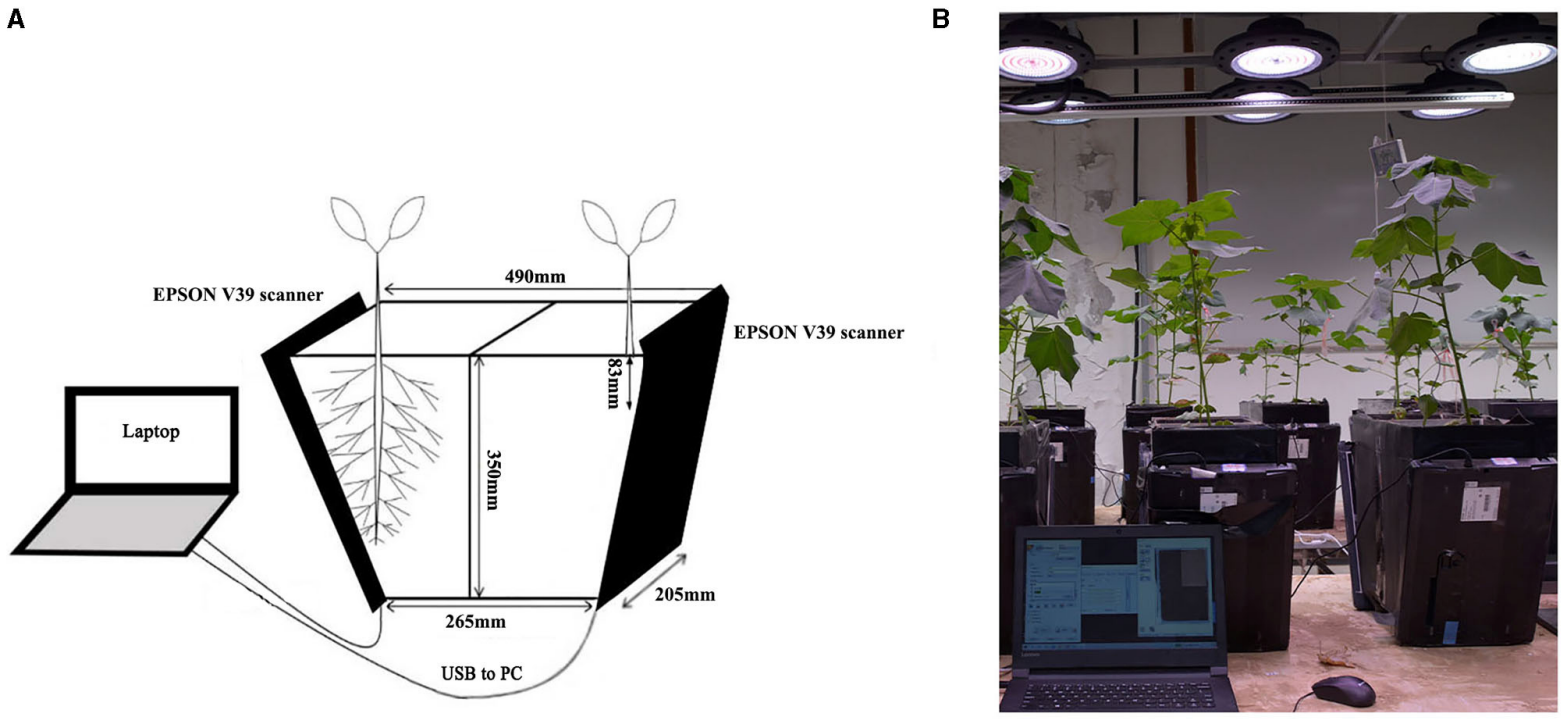
Schematic representation **(A)** and image **(B)** of the RhizoPot growth-imaging device.

Two levels of P, 0 mg/kg (P-deficient, PD) and 130 mg/kg (P-replete, PR), (Klamer et al., 2019) were tested. The experiment consisted of six replicates. RhizoPot (Figure 1), an improvised *in situ* root device, was used as the culture device (Xiao et al., 2020). The RhizoPot contained 15 kg of a soil mixture. The soil was sampled from the experimental cotton field of Hebei Agricultural University at a depth of 40–60 cm. The soil had the following characteristics: pH 7.20; organic matter content, 2.9 g/kg; total N, 0.14 g/kg; alkali-hydrolyzable N, 7.56 mg/kg; available P, 3.09 mg/kg; available potassium, 74.67 mg/kg; and soil and sand, 4:1 (*w/w*).

The seedlings were maintained at 26/20°C (day/night) and 45–50% relative humidity with a 14/10 h (day/night) photoperiod (600 μmol/m^2^/s^1^ light intensity). The seedling culture medium was supplemented with nitrogen (urea, N, 46.7%) and potassium (potassium sulfate, K_2_O, 50%) at concentrations of 105 mg/kg and 53.3 mg/kg, respectively (Geng et al., 2020).

Seeds were surface sterilized by soaking in 75% (v/v) ethyl alcohol solution for 15 min followed by three washes with sterile distilled water. The seeds were then incubated at 25°C for 24 h in an incubator to remove unterminated seeds. The germinated seeds were sown 3 cm from the scanner panel of the RhizoPot at a depth of 3 cm when the relative soil moisture content was 70%.

### Measurement of Morphological Parameters and Photosynthetic Indicators

Crop phenotypic parameters and photosynthetic indicators were determined at 30, 40, and 50 days after sowing. Plant height and stem diameter were measured using a ruler and vernier calipers. Leaf area was calculated using the length–width coefficient method (Mao et al., 2014). The chlorophyll concentrations of the third functional leaves from the top were determined using a SPAD meter (SPAD-502; Konica Minolta, Tokyo, Japan).

Six representative cotton plants from each RhizoPot were used to measure the net photosynthetic rate (Pn). The measurements were conducted using an LI-6400 portable photosynthesis system (LI-COR, Lincoln, NE, USA) under ambient conditions (irradiation, 600 μmol/m^2^/s^1^; leaf temperature, 25 ± 1°C) from 10 to 11 a.m. The maximum photochemical efficiency (*F*_v_/*F*_m_) was measured using a portable FMS-2 fluorometer (Hansatech, King's Lynn, UK) at the same time as Pn measurements.

### Measurement of Biomass and P Content

Plants were harvested 50 days after sowing and cut off at the cotyledon node to separate the shoots from the roots. The shoots and roots were dried at 85°C for 48 h and then weighed to obtain the dry weight and root/shoot (R/S) ratio. The dried samples were then ground and sieved. The P content (PC, g/kg) of stems and roots was determined using the vanadium molybdenum yellow colorimetric method.

### Root Image Collection

Scanning was performed using a scanner (Epson Perfection V39, Suwa, Japan) attached to the RhizoPot device as described by Xiao et al. (2020). *In situ* images of the root system were obtained daily starting at 8 a.m. Scanning was performed to obtain *in situ* root images at 1,200 dpi and 4,800 dpi resolutions. The images were then analyzed to obtain root and root hair phenotype data.

A deep learning tool (DeepLabv3+) was used to segment the *in situ* root image at a resolution of 1,200 dpi (Shen et al., 2020). With this tool, the root system was shown in white, and the culture medium was shown in black. The segmented root pictures were analyzed using WinRHIZO REG2009 (Regent Instruments, Inc., Quebec City, Canada). The average daily growth of taproots (ADGT, cm), the average root diameter (AD, mm), the net growth rate per unit volume (RLD_NGR_, cm/cm^3^/day), and LRN were calculated based on the *in situ* root collection frequency. The root length density per unit volume (RLD, cm/cm^3^) and the LRD (branching/cm^1^) were calculated using the above parameters as follows (Johnson et al., 2001):

$$\begin{array}{l} \text{RLD}=\text{RL}/\text{A}\times \text{DOF}\\ \text{LRD}=\text{ LRN}/\text{TRL}. \end{array}$$

Here, A (cm^2^) is the area of the observation window, DOF (cm) is the observable soil thickness (0.25 cm) (Oosterveld et al., 1985; Steele et al., 1997), and TRL (cm) is the taproot length.

### Estimation of Root Hair Length and Life Span

The root hair phenotype data extracted from the root image were analyzed using Adobe Photoshop CC 2019 (Adobe, San Jose, CA, USA). Root hair length was measured using the “Ruler Tool” in the software. A total of 50 images were taken per sample. Adobe Photoshop was used to open different time-series images of the same RhizoPot to observe root hair life span (RHL). Root hair longevity was determined as described by Xiao et al. (2020).

### Specific Root Length and P Uptake Measurements

Specific root length (SRL) and P uptake (PU) were measured using the formulae mentioned below:

$$\begin{array}{l} \text{ SRL}\left(\text{m}/\text{g}\right)=\text{total root length}/\text{root dry weight}..\\ \text{PU}\left(\text{mg}/\text{plant}\right)=\text{PC}\times \left(\text{shoot dry weight }+\text{ root dry weight}\right). \end{array}$$

### Statistical Analyses

One-way ANOVA was performed using IBM SPSS Statistics 26.0 (IBM Corp., Armonk, NY, USA). Differences between treatments were considered significant according to Duncan's test at a *P* < 0.05 threshold. Survival analysis was performed using the Kaplan–Meier method (Kaplan and Meier, 1958). The average life span of the root hairs was the average of the survival days. The median RHL (i.e., the time at which the survival rate reached 50%) was estimated, and survival curves were generated (Xiao et al., 2020). Figures were drawn using GraphPad Prism 8 (GraphPad Software Inc., San Diego, CA, USA). Principal component analysis (PCA) was performed using R software (R 4.0.4).

## Results

### Effect of Low P Stress on Cotton Shoot Morphology

**Figure 2:**
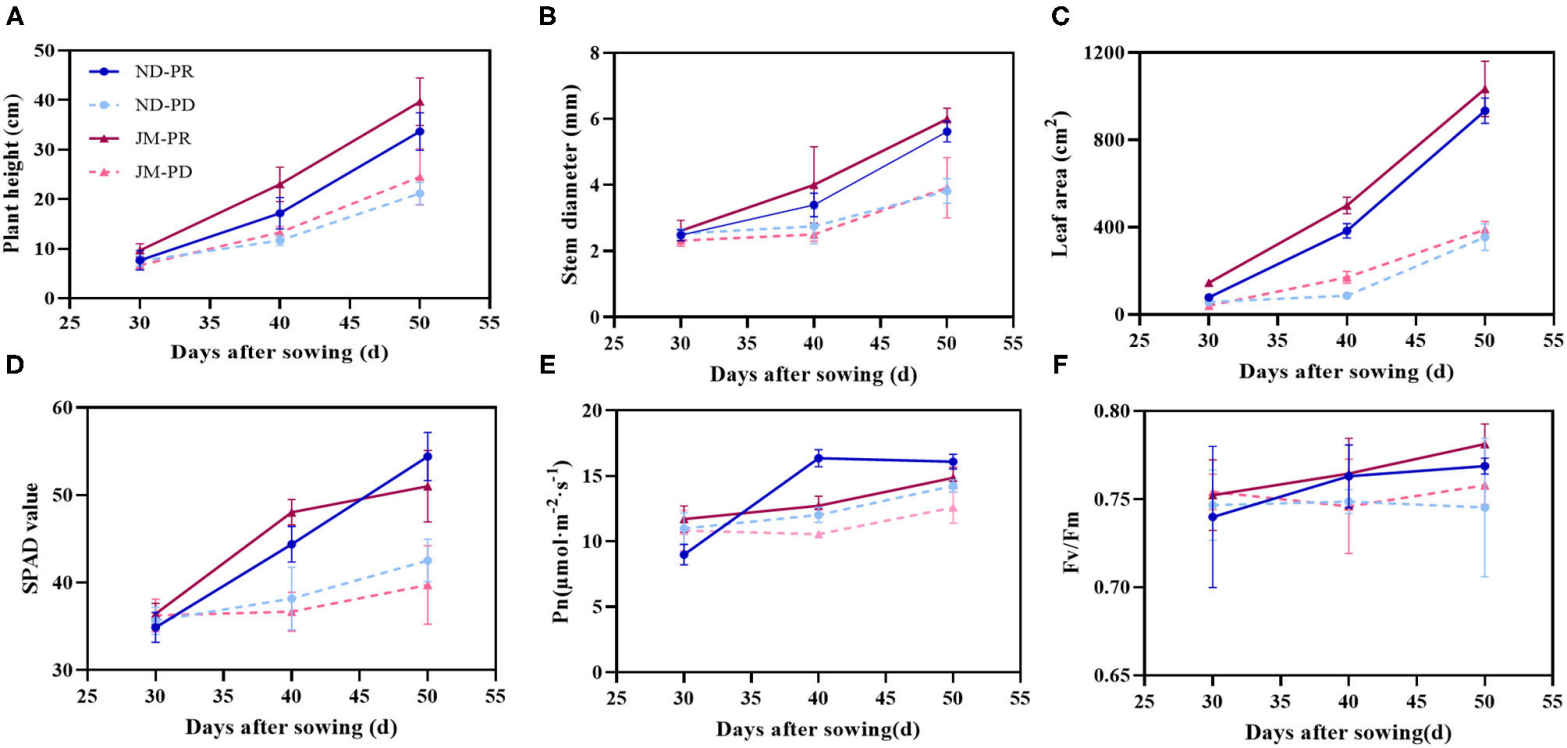
Seedlings of two cotton cultivars grown under P-replete (PR) and P-deficient (PD) conditions for 50 days. Plant height **(A)**, stem diameter **(B)**, leaf area **(C)**, SPAD value **(D)**, net photosynthetic rate (Pn) **(E)**, and the maximum photochemical efficiency (*F*_v_/*F*_m_) **(F)**. ND-PR, “Nongdamian No. 10” cultivar under replete phosphorus conditions; ND-PD, “Nongdamian No. 10” cultivar under low phosphorus; JM-PR, “Jimian 315” cultivar under replete phosphorus conditions; JM-PD, “Jimian 315” cultivar under low phosphorus. Depicted are the means of six replicates ± standard errors.

Aboveground phenotypes of cotton seedlings, including plant height, stem diameter, leaf area, and SPAD value, increased during the initial culture period under P-deficient (PD) conditions (Figures 2A–D). However, at 40 and 50 days after sowing (DAS), the cotton growth under PD conditions was significantly lower than that under P-replete (PR) conditions (Supplementary Figure 1). At 50 DAS, plant height, stem diameter, leaf area, and SPAD value of ND plants under PD conditions were reduced by 37.13, 32.05, 62.05, and 21.80%, respectively, compared with that under PR conditions. The plant height, stem diameter, leaf area, and SPAD value of JM plants under PD conditions were 38.24, 34.72, 62.36, and 22.12% lower than those of JM plants under PR conditions, respectively.

Phosphorus-deficient treatment decreased Pn and *F*_v_/*F*_m_ in cotton (Figures 2E,F). The maximum difference in Pn between the PD and PR treatments was a 26.46% decrease in Pn, which occurred at 40 DAS under the PD treatment. PD conditions did not significantly affect *F*_v_/*F*_m_ at 30 DAS. However, PD treatment significantly decreased the *F*_v_/*F*_m_ of cotton as the cultivation period continued. At 50 DAS, the *F*_v_/*F*_M_ of ND and JM plants under the PD treatment was 3.06 and 2.99% lower than that under the PR treatment.

### Effect of Low P Stress on Cotton Dry Weight, R/S Ratio, SRL, and PU

**Figure 3:**
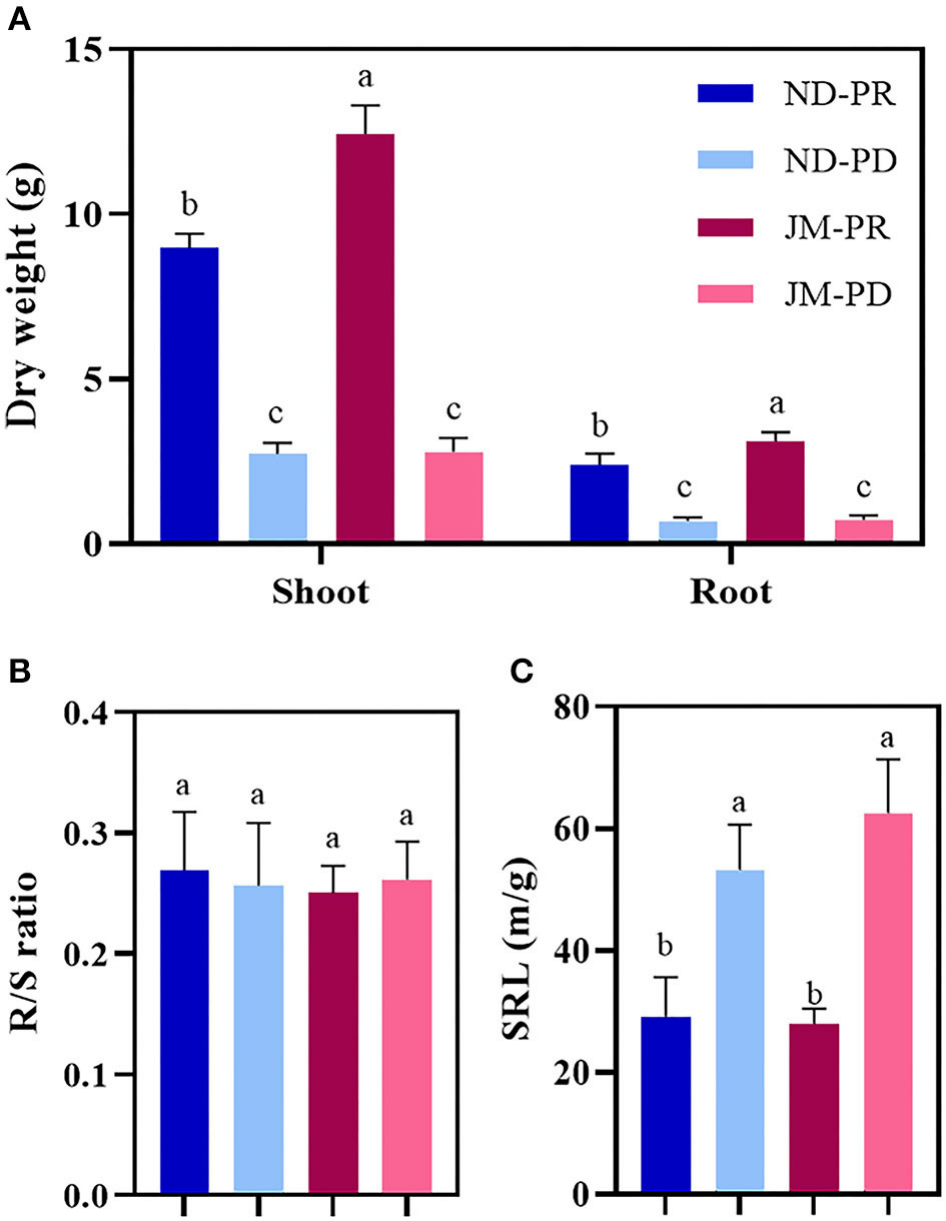
Effect of phosphorus stress on shoot and root dry weight of two cotton cultivars **(A)**, root/shoot ratio (R/S ratio) **(B)**, and specific root length (SRL) **(C)**. ND-PR, “Nongdamian No. 10” cultivar under replete phosphorus conditions; ND-PD, “Nongdamian No. 10” cultivar under low phosphorus; JM-PR, “Jimian 315” cultivar under replete phosphorus conditions; JM-PD, “Jimian 315” cultivar under low phosphorus. Depicted are the means of six replicates ± standard errors. For each trait, bars with the same letter are not significantly different according to Duncan's test at a *P* < 0.05 threshold.

Cotton was harvested at 50 DAS to measure the shoot dry weight and root dry weight (RDW). The PD condition significantly reduced the dry weight of cotton shoot and root tissue (Figure 3A). The shoot dry weights of ND and JM plants under PD conditions were 69.70 and 77.45% lower than those under PR conditions. The RDW of JM under PR conditions was 4.35 times higher than that under PD conditions. The R/S ratios of ND and JM plants under PD conditions were 4.71 and 4.31% lower than those under PR conditions (Figure 3B). PD conditions significantly increased the SRL (Figure 3C). The SRLs of ND and JM plants under PD conditions were increased by 82.70 and 123.62%, respectively, compared with PR conditions.

**Figure 4:**
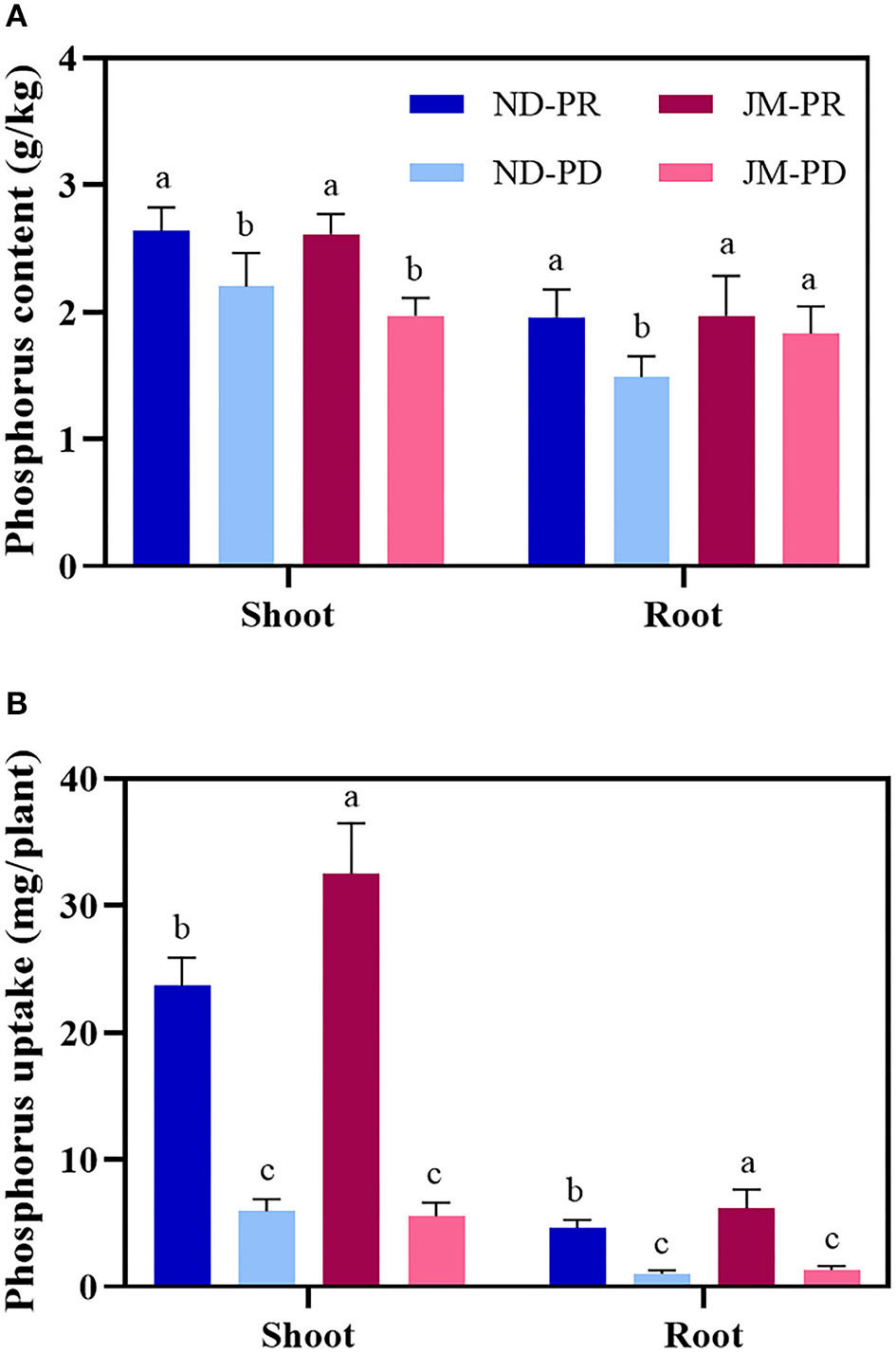
Phosphorus content **(A)** and uptake **(B)** in root and shoot tissues of two cotton cultivars under different phosphorus levels. ND-PR, “Nongdamian No. 10” cultivar under replete phosphorus conditions; ND-PD, “Nongdamian No. 10” cultivar under low phosphorus; JM-PR, “Jimian 315” cultivar under replete phosphorus conditions; JM-PD, “Jimian 315” cultivar under low phosphorus. Depicted are the means of six replicates ± standard errors. For each trait, bars with the same letter are not significantly different according to Duncan's test at a *P* < 0.05 threshold.

Phosphorus-deficient conditions significantly reduced the PCs of cotton shoots at 50 DAS (Figure 4A). Compared with PR conditions, the PC of ND and JM aboveground biomass under PD conditions decreased by 16.69 and 24.56%, respectively. PD conditions significantly reduced the PC in the ND root system. ND plants under PD conditions were reduced by 24.07% compared with that under PR conditions, whereas PD conditions had a negligible effect on JM plants. PD conditions significantly reduced the PU of cotton plants (Figure 4B). Compared with ND plants under PR conditions, the shoot and root biomass PU under PD conditions decreased by 74.86 and 78.49%, respectively. Compared with JM plants under PR conditions, the PU of the shoots and roots under PD conditions decreased by 82.94 and 78.93%, respectively.

### Effect of Low P Stress on ADGT, Net Growth Rate Per Unit Volume (RLD_NGR_), and Root Length Density Per Unit Volume (RLD)

**Figure 5:**
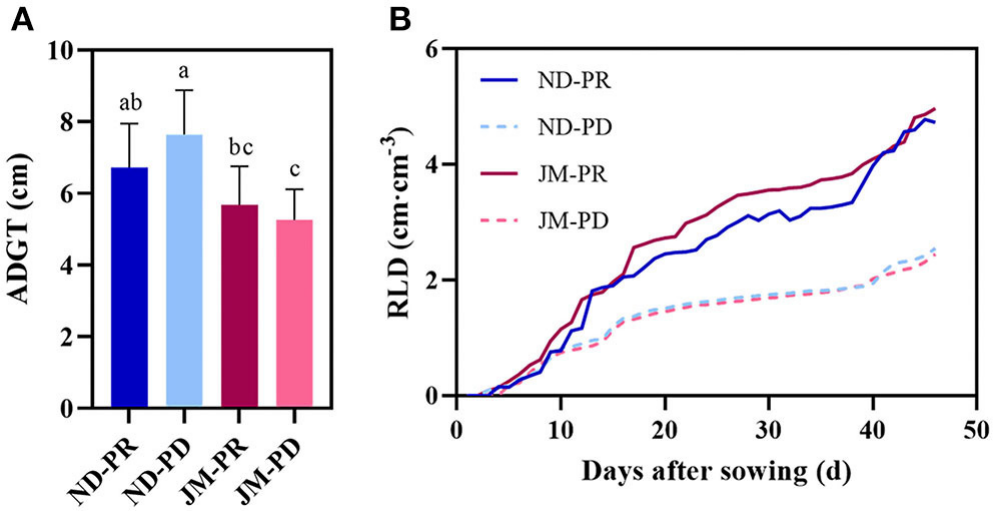
Difference in average daily growth of taproots (ADGT) **(A)** and lateral root branching density per unit volume (RLD) **(B)** of two cotton cultivars under different phosphorus levels. ND-PR, “Nongdamian No. 10” cultivar under replete phosphorus conditions; ND-PD, “Nongdamian No. 10” cultivar under low phosphorus; JM-PR, “Jimian 315” cultivar under replete phosphorus conditions; JM-PD, “Jimian 315” cultivar under low phosphorus. Depicted are the means of six replicates ± standard errors. For each trait, bars with the same letter are not significantly different according to Duncan's test at a *P* < 0.05 threshold.

The difference in ADGT between the two cotton varieties under different P levels was not significant (Figure 5A). The ADGT of ND plants rose substantially under PD conditions, increasing by 13.58% compared with PR conditions. The difference in ADGT for JM plants between the two P levels was small and only reduced by 7.4% under PD conditions compared with PR conditions, indicating that the ADGT of JM plants was not sensitive to P deficiency.

After observing the growth and development of the two cotton cultivars for 50 days under different P levels, variation in RLD_NGR_ was noted (Table 1). In the early stage of observation, RLD_NGR_ was the highest at 10 DAS. This was also the highest peak of daily growth of the two varieties under PD conditions. The RLD_NGR_ of ND and JM plants were 3.49 and 3.83 cm, respectively, at 10 DAS. After day 10, RLD_NGR_ began to decline gradually in both cultivars. The RLD_NGR_ values of ND and JM plants were lowest at 34 DAS, that is, 0.49 and 0.55 cm, respectively. RLD_NGR_ began to increase from the 40th day onward. Significant differences were observed between different P levels over the late observation period. During days 40–46, the total root growth of ND and JM plants under PD conditions decreased by 59.9 and 48.0%, respectively, compared with those of PR conditions. During the entire observation period, the RLD_NGR_ values of the two cotton cultivars were positive irrespective of the P levels.

**Table 1:** Difference in the net growth rate per unit volume (cm/cm^3^/day) of cotton under different P levels.

**Days after sowing**	**ND-PR**	**ND-PD**	**JM-PR**	**JM-PD**
4d	1.13 ± 0.26a	1.08 ± 0.18a	1.11 ± 0.14a	0.00 ± 0.00b
10d	5.02 ± 0.34a	3.49 ± 0.29b	5.19 ± 1.41a	3.83 ± 0.19b
16d	4.89 ± 0.29a	2.67 ± 0.32b	4.9 ± 0.54a	2.73 ± 0.26b
22d	2.31 ± 0.24b	1.27 ± 0.12c	4.51 ± 0.35a	1.28 ± 0.34c
28d	2.64 ± 0.45a	0.67 ± 0.12b	2.26 ± 0.17a	0.66 ± 0.26b
34d	1.07 ± 0.55a	0.49 ± 0.16b	0.81 ± 0.25ab	0.55 ± 0.20b
40d	4.94 ± 0.39a	0.68 ± 0.37d	2.28 ± 0.37b	1.35 ± 0.23c
46d	4.10 ± 0.73a	3.10 ± 0.21b	4.52 ± 0.53a	2.18 ± 0.41c

*P, phosphorus; ND-PR, “Nongdamian No. 10” cultivar under replete P conditions; ND-PD, “Nongdamian No. 10” cultivar under low P; JM-PR, “Jimian 315” cultivar under replete P conditions; JM-PD, “Jimian 315” cultivar under low P*.

*Depicted are the means of six replicates ± standard error. For each trait, bars with the same letter are not significantly different according to Duncan's test at a P < 0.05 threshold*.

After 2 weeks of treatment with different P levels, significant differences were observed in RLD between the two cotton varieties. RLD was significantly reduced in the later stages of cotton growth under PD conditions (Figure 5B). The maximum RLD difference (49.31%; 2.36 cm/cm^3^) between ND plants under PD and PR conditions was observed at 45 DAS. The maximum difference in RLD (53.87%; 2.59 cm/cm^3^) between JM plants under PD and PR conditions was observed at 44 DAS.

### Effects of Low P Stress on the LRN, LRD, and AD

**Figure 6:**
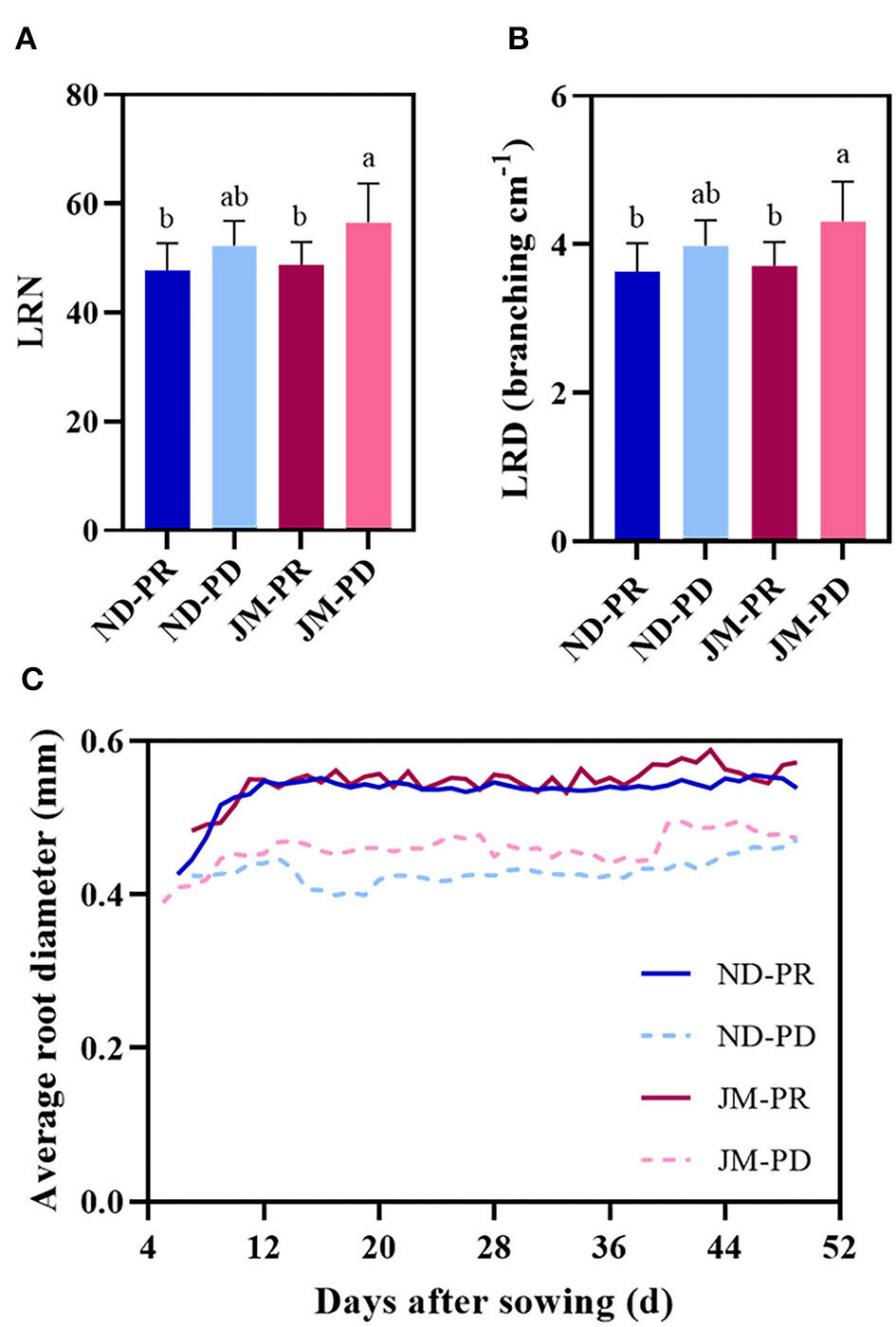
Trends in the number of lateral roots (LRN) **(A)**, lateral root branching density (LRD) **(B)**, and average root diameter (AD) **(C)** of two cotton cultivars under different phosphorus levels. ND-PR, ‘Nongdamian No. 10” cultivar under replete phosphorus conditions; ND-PD, “Nongdamian No. 10” cultivar under low phosphorus; JM-PR, “Jimian 315” cultivar under replete phosphorus conditions; JM-PD, “Jimian 315” cultivar under low phosphorus. Depicted are the means of six replicates ± standard errors. For each trait, bars with the same letter are not significantly different according to Duncan's test at a *P* < 0.05 threshold.

Phosphorus-deficient conditions significantly increased the LRN and LRD of the cotton root system (Figures 6A,B). Compared with ND plants under PR conditions, the LRN and LRD of ND plants under PD conditions increased by 9.44%. Regarding the cultivar JM, the LRN and LRD were 16.1% higher under PD conditions than that under PR conditions, indicating that PD conditions can promote the growth and development of lateral roots in cotton. The differences between LRD and LRN showed the same trend throughout the experimental period.

Phosphorus-deficient conditions significantly reduced the AD of lateral roots (Figure 6C). The lateral roots were relatively thin (0.40 mm) when they first appeared but broadened with time. Under PR conditions, the AD of lateral roots fluctuated in the range of 0.50–0.60 mm, whereas under PD conditions, the range was between 0.40 and 0.45 mm. The diameter of lateral roots gradually increased under PD conditions and ultimately stabilized between 0.45 and 0.50 mm.

### Effect of Low P Stress on Average Root Hair Length and RHL

**Figure 7:**
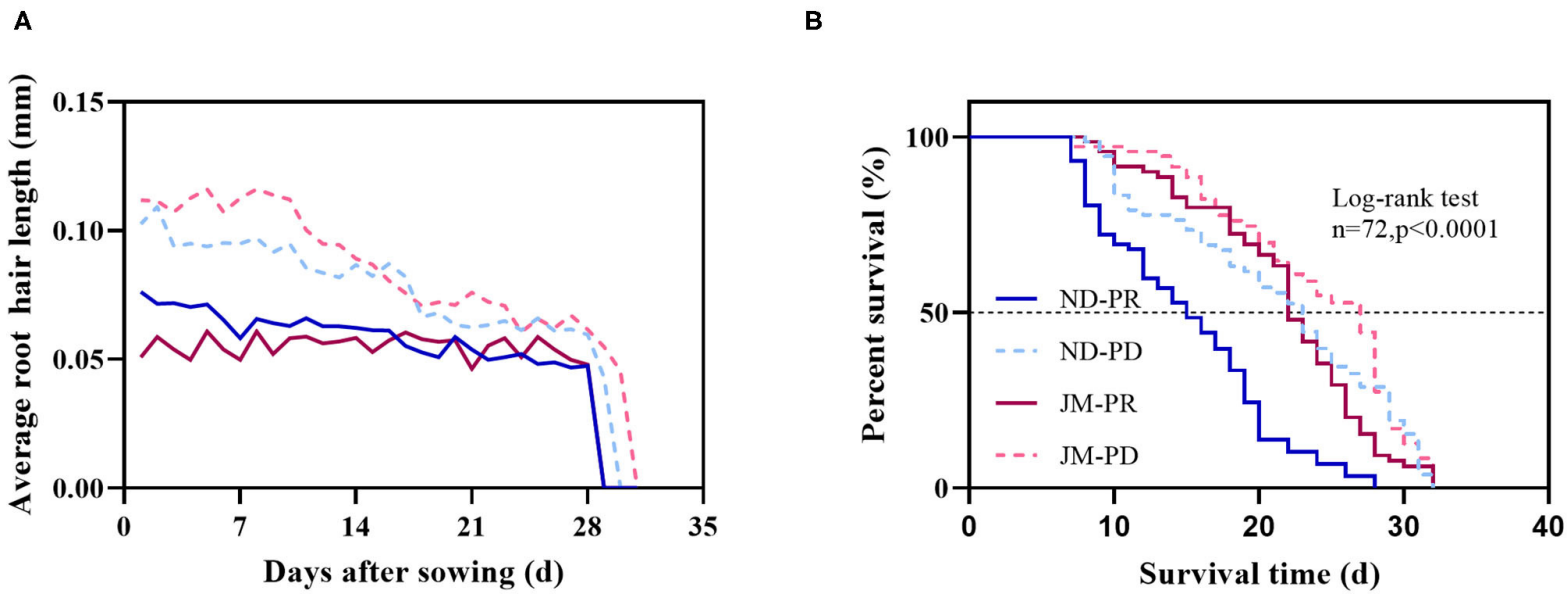
Effect of phosphorus stress on the average root hair length **(A)** and root hair life span **(B)** after 50 days of phosphorus treatment. ND-PR, “Nongdamian No. 10” cultivar under replete phosphorus conditions; ND-PD, “Nongdamian No. 10” cultivar under low phosphorus; JM-PR, “Jimian 315” cultivar under replete phosphorus conditions; JM-PD, “Jimian 315” cultivar under low phosphorus; *n*, the number of root hair used to draw the curve. The *P*-values indicate the statistical significance of phosphorus stress on the median life span (days) of cotton plants.

Phosphorus-deficient conditions significantly increased the average root hair length (ARHL) of both cotton cultivars (Figure 7A). The difference in ARHL between the two P levels was highest at the early stage of root hair growth for both cultivars; however, the difference gradually decreased after 2 weeks. Under the same level of P treatment, the ARHL of JM plants was higher than that of ND plants by up to 125.85%. Notably, the root hairs of JM plants were more sensitive to PD conditions. These results show that PD conditions significantly increased ARHL.

**Figure 8:**
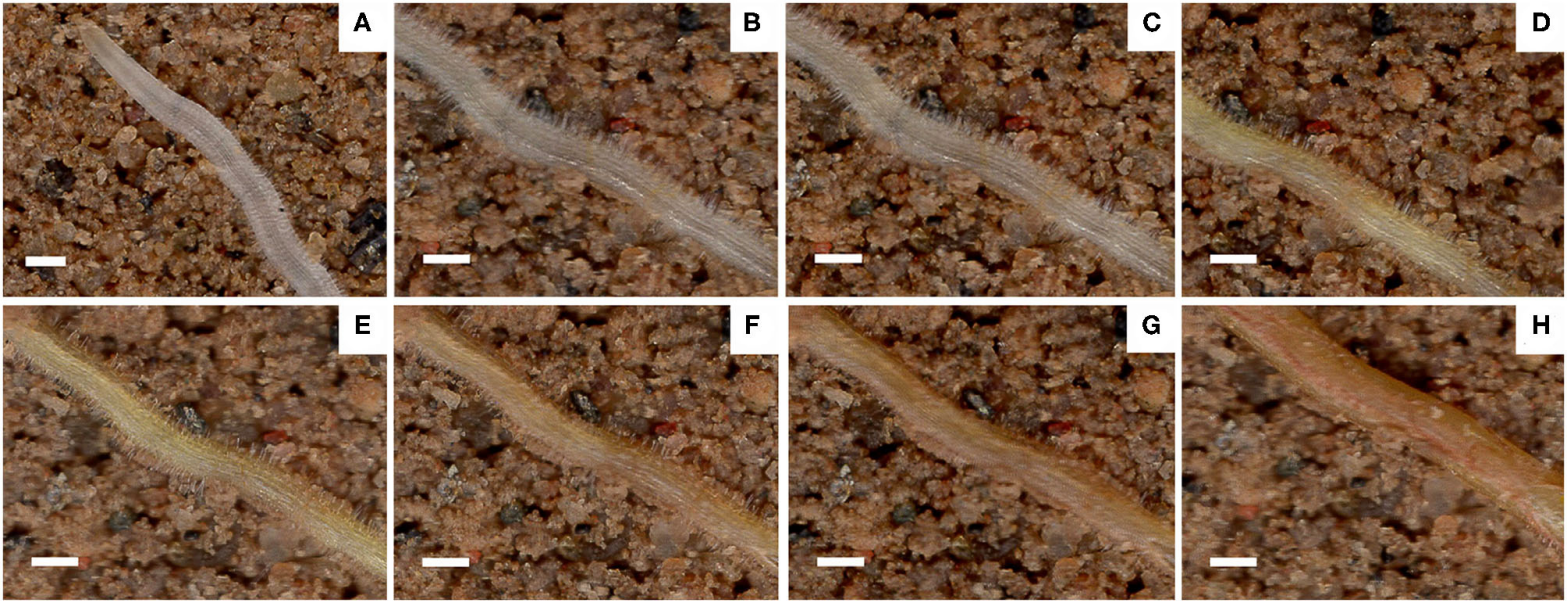
Images of the same root area taken at different times during the life span of cotton root hairs. Scale bar, 0.5 mm. Images shown are taken on **(A)** 30 November, **(B)** 1 December, **(C)** 6 December, **(D)** 12 December, **(E)** 18 December, **(F)** 24 December, **(G)** 30 December, and **(H)** 5 January.

Root hair life span is the period between the appearance of root hair and the end of its appearance distortion (Figure 8). The median root hair life of ND plants under PR and PD conditions was 15 and 23 days, while that of JM plants was 22 and 27 days, respectively (Figure 7B). PD conditions significantly prolonged the root hair life of both cotton cultivars. Notably, the root hairs of ND plants were more sensitive to low P stress than those of JM plants. These results indicate that low P stress significantly increases RHL.

### Correlation and Principal Component Analyses

**Figure 9:**
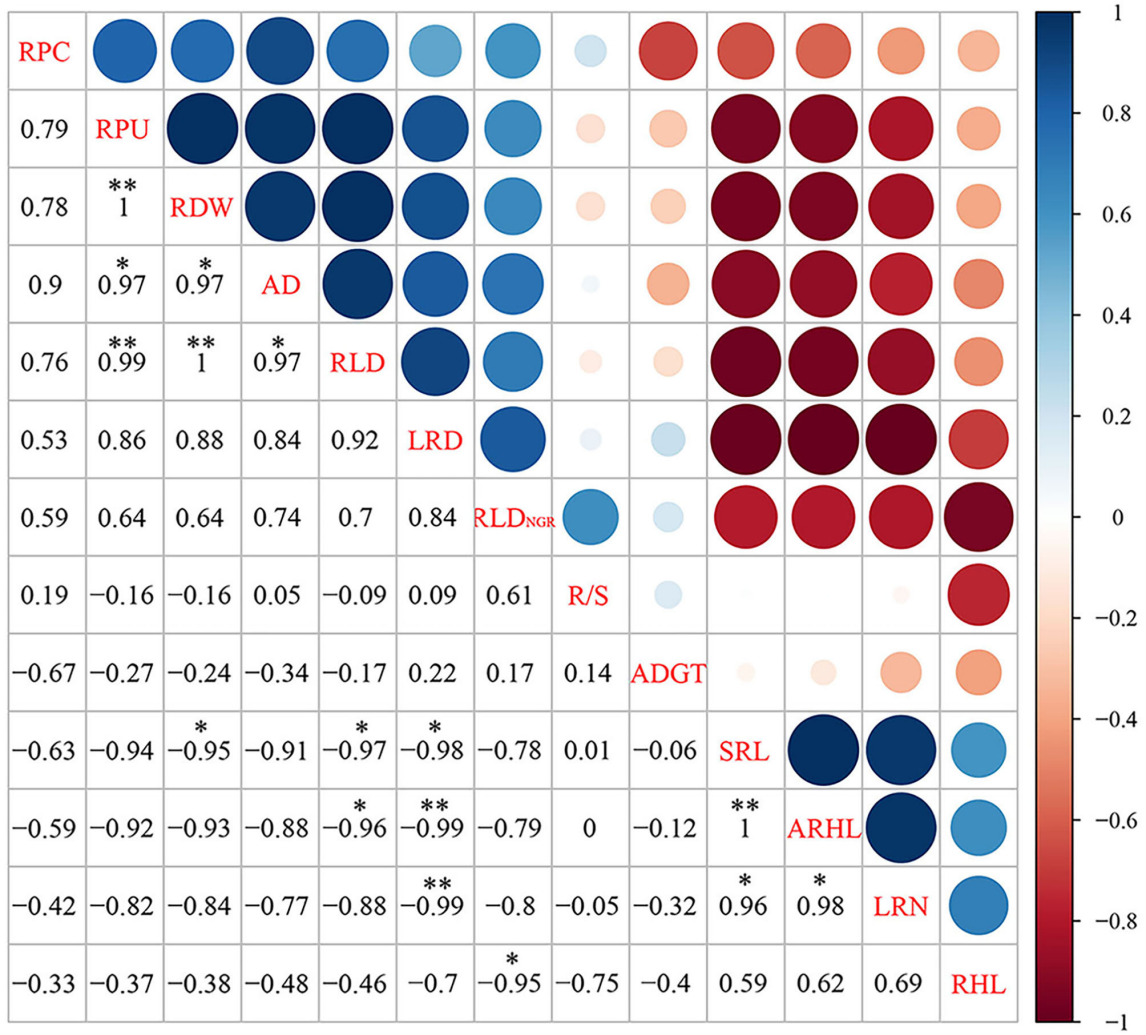
The matrix of Spearman's correlation coefficients and 95% confidential intervals among root and shoot morphology indices. The significance level of correlations is indicated as follows: *P < 0.05; **P < 0.01. RPC, root phosphorus content; RPU, root phosphorus uptake; RDW, root dry weight; R/S, root/shoot ratio; ADGT, average daily growth of taproots; RLD_NGR_, net growth rate per unit volume; RLD, root length density per unit volume; LRN, number of lateral roots; LRD, lateral root branching density; AD, average root diameter; ARHL, average root hair length; RHL, root hair life span; SRL, specific root length.

A significant positive correlation was observed between root PU (RPU), RDW, RLD, and AD (Figure 9). RPU had a significant positive correlation with RDW and RLD, whereas RDW had a significant positive correlation with RLD. There was a significant positive correlation between SRL, ARHL, and LRN, and a significant positive correlation between SRL and ARHL. SRL was negatively correlated with RDW, RLD, and LRD. LRD had significant negative correlations with ARHL and LRN. There was a significant negative correlation between RHL and RLD_NGR_. No significant correlation was observed between RPC, R/S, ADGT, and other indicators.

**Figure 10:**
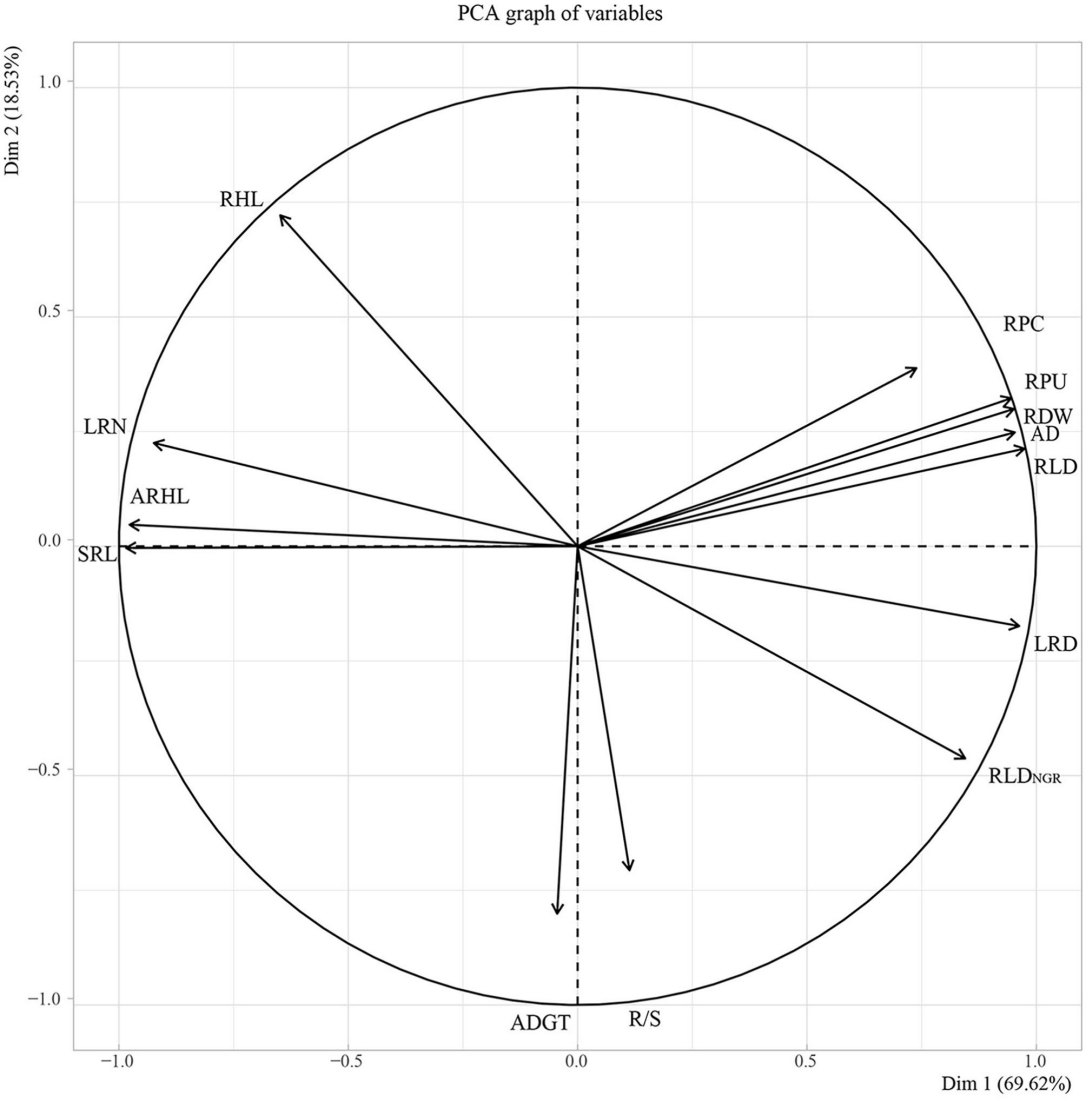
Principal component analysis (PCA) of 11 root *in situ* traits. RPC, root phosphorus content; RPU, root phosphorus uptake; RDW, root dry weight; R/S, root/shoot ratio; ADGT, average daily growth of taproots; RLD_NGR_, the net growth rate per unit volume; RLD, root length density per unit volume; LRN, number of lateral roots; LRD, lateral root branching density; AD, average root diameter; ARHL, average root hair length; RHL, root hair life span; SRL, specific root length.

Subsequently, PCA was performed on root traits (Figure 10). The contribution rate of the first two principal components was 88.15%. Among them, principal component one contributed 69.62%, with LRD, RLD, RDW, AD, RLD_NGR_, and RPU contributing to principal component one. Principal component two contributed 18.53%, with ARHL, SRL, LRN, and ADGT, R/S contributing to axis one and axis two but in opposite directions.

## Discussion

### Low P Stress Inhibits the Growth of Cotton Shoots

Soil PC is one of the main restrictive factors for crop growth and development. P deficiency impacts significant changes in the morphology of aboveground plant parts. For example, a lack of P reduces the photosynthetic rate and inhibits metabolism. These changes cause slow growth and development of plants, resulting in shorter plants with smaller leaf areas (Jacob and Lawlor, 1991; Lima et al., 1999, 2000). Low P stress reduces the leaf area index and photosynthetic rate of rice (Deng et al., 2020). Studies on corn, wheat, rapeseed, and broad bean suggest that low P stress can inhibit the growth of aboveground plant parts. In this study, low P stress significantly reduced the plant height, main stem thickness, leaf area, SPAD, Pn, and *F*_v_/*F*_m_ values of cotton (Figure 2). Among these parameters, the leaf area, which has the most significant impact on the shoot phenotype, was the most affected by low P stress. Compared with PR conditions, the leaf area of cotton ND and JM cultivars decreased by 62.05 and 62.36%, respectively, indicating that the long-term low P stress can inhibit the growth and development of cotton shoots (Supplementary Figure 1). A study by Li et al. (2020). on cotton also concluded that low P stress reduces plant height and leaf area.

### Low P Stress Reduces Cotton Dry Matter Weight, PC, P Absorption, and SRL

The stems and leaves constitute the aboveground biomass of plants at the seedling stage (Chen et al., 2012). Low accumulation of photosynthetic products suppresses plant height and leaf area, thus reducing the total dry weight of the aerial parts. In low P soils, nutrient transportation to the surface of cotton roots limits the absorption of P by the roots (Rosolem et al., 1999).

Low P stress increases the respiratory load of root cells and biomass distribution to roots and significantly reduces the aboveground biomass. These changes collectively decrease the total plant dry weight (Nielsen et al., 2001). In this study, the difference in cotton dry matter accumulation between PD conditions and the normal P treatment gradually increased after 30 days (Figure 3A). The total dry weight of cotton reduced by 70.04 and 77.26% in the ND and JM cultivars, respectively, under low P stress, compared with PR conditions. These findings are consistent with the results of Xie et al. (2021), who found that low P stress can significantly reduce the dry weight of cedar trees.

Low P stress significantly affects the SRL of plants. The SRL of chickpea plants was longer under low P stress (Wang et al., 2021). Bamboo also gains more P by increasing SRL and thus its foraging range (Yang et al., 2021). Grasses perform better under low P stress than that under sufficient P conditions because they possess longer roots, which maximizes their P utilization (Tshewang et al., 2020). Studies on peppers have shown that low P stress enhances root growth by increasing the total root length and SRL (Pereira-Dias et al., 2020). In this study, low P stress significantly increased the SRL of both cotton cultivars (Figure 3C). Specifically, the optimum P for ND and JM cultivars increased by 82.70 and 123.62%, respectively.

Under sufficient P levels, the adsorption saturation of P in the soil increases. In turn, the effective PC of the soil increases significantly, leading to higher levels of available P (Shao et al., 2006). Plants usually maximize P absorption by increasing the root length and root surface area under low P stress (Zhang et al., 2013). Studies show that regardless of P levels in the soil, the more P accumulates in the ground, the larger the root system and the better the root structure (Zhu et al., 2010). Studies on wheat suggest that P absorption decreases with a decrease in RDW (Soumya et al., 2021). In this study, PC and P absorption were directly proportional to shoot dry weight and RDW (Figure 3A). Low P stress significantly reduced the PC and P absorption of shoots and roots (Figure 4). Under low P stress, the PCs of ND and JM plants were reduced by 19.83 and 17.18%, respectively, compared with PR conditions. The total P absorption of cotton was reduced by 75.45 and 82.30% in ND and JM cultivars, respectively, under low P stress, compared with PR conditions. In this study, the amount of P absorbed by roots was positively correlated with the AD, RDW, and RLD (Figure 9). These results are similar to those reported by Duan et al. (2020), who concluded that PC increases with the increase in RDW. Yan et al. (2019) found that the root biomass and average diameter in P-poor patches were thinner and longer. Thus, root growth patterns can change to adapt to lower P availability in soil.

### Effect of Low P Stress on Cotton Root Phenotypes

The root system is an important organization for plants to explore and obtain P from the soil. P has a low mobility and utilization rate in the soil, and the amount of P obtained mainly depends on the root phenotype (Yano and Kume, 2005). The average diameter of the root system significantly influences P absorption by crops (Lugli et al., 2021). Notably, the exogenous application of P usually increases the diameter of the root system. Reducing the root diameter is an effective and economical way of increasing the availability of P because it reduces the consumption of photosynthetic products (Lynch, 2011, 2013). In this study, low P stress significantly reduced the root diameter (Figure 6C). The AD of roots under PR conditions fluctuated in the range of 0.50–0.60 mm, whereas the AD under PD conditions was 0.40–0.50 mm. There were significant correlations between both root diameter and PU as well as RDW and RLD_NGR_ (Figure 9). The reduction in AD could be owing to the formation of secondary lateral roots at the particular growth period (Table 1).

The formation of lateral roots requires fewer photosynthetic products than other types of roots (Wang et al., 2016). Thus, plant varieties with more branched roots are better adapted to low P stress (Zhu and Lynch, 2004). Studies on *Arabidopsis* and rice show that low P stress induces the development of lateral roots, and increasing the LRN can significantly increase the absorption capacity of P (Li et al., 2001; Fitter et al., 2002). In this study, compared with PR conditions, the LRN and LRD of ND and JM plants under low P increased by 9.44 and 16.1%, respectively (Figures 6A,B), indicating that cotton roots could adapt to low P stress. The results showed that the aboveground plant phenotypes of the two cotton varieties were similar, and the response trend of their roots to low P was the same, but the response degree was different. This indicated that the tolerance of the two cotton varieties to low P was different, which was also the reason for the selection of the two varieties in this study.

The net growth rate of the root system is an important indicator of its development and senescence. In this study, the RLD_NGR_ in the two cotton cultivars was positive (Table 1), indicating that the growth rate of the root system was greater than the senescence rate. This is because the vegetative growth of cotton mainly occurs during the seedling stage, and the plants form more new lateral roots at this stage.

### Low P Promotes Root Hair Elongation and Delays Root Hair Senescence

The root system is in direct contact with the soil and is, therefore, the initial sensor of low P stress (Bates and Lynch, 2001). Increasing the length of root hairs is an adaptive strategy of plants under low P stress (Cao et al., 2013). In citrus, the length of root hairs increases significantly under low P stress, whereas in *Arabidopsis*, the increase is mainly observed with a prolonged period of low P stress (Bates and Lynch, 1996). In this study, the length of root hairs under low P stress was longer than that under sufficient P conditions (Figure 7A). However, the phenotype of the aboveground parts did not differ between P treatments until 30 DAS (Figure 2), indicating that the root hairs respond to low P stress much earlier than the shoots. The root hair lengths of ND and JM cultivars increased by 32.96 and 56.87%, respectively, under low P stress compared with the sufficient P treatment, indicating that low P stress can promote root hair elongation. The ARHL was significantly correlated with the SRL, LRN, LRD, and RLD, but not with the PU (Figure 9). In this study, the length of root hairs affected the morphological characteristics of the root system but did not influence PU. This is contrary to the conclusions of Brown et al. (2012), who found that low P stress and root hair length can increase the accumulation, biomass, and yield of the aboveground P in barley. However, Brown et al. compared a variety without root hairs with one that has root hairs. Therefore, they concluded that the presence of root hairs has a significant impact on plant PU.

Root hairs have a short life span and typically degenerate within 10–20 days. However, within a specific range of P levels, low P stress can enhance root hair development. For example, in this study, the average life span periods of root hairs in ND and JM cultivars were 8 and 5 days longer under PD conditions compared with PR conditions. Both genetic and environmental factors influence root hair longevity (Shi and Zhu, 2002). In this study, genotypic differences in the longevity of the root hairs were observed between the two cotton cultivars. JM plants exhibited a longer RHL than ND plants under similar P conditions (Figure 7B), which can be attributed to genetic differences between the two varieties. Regarding environmental influence, low P stress prolonged the life span of ND and JM root hairs by 8 and 5 days, respectively, compared with PD conditions, indicating that low P stress can significantly improve the RHL of cotton seedlings.

## Conclusion

**Figure 11:**
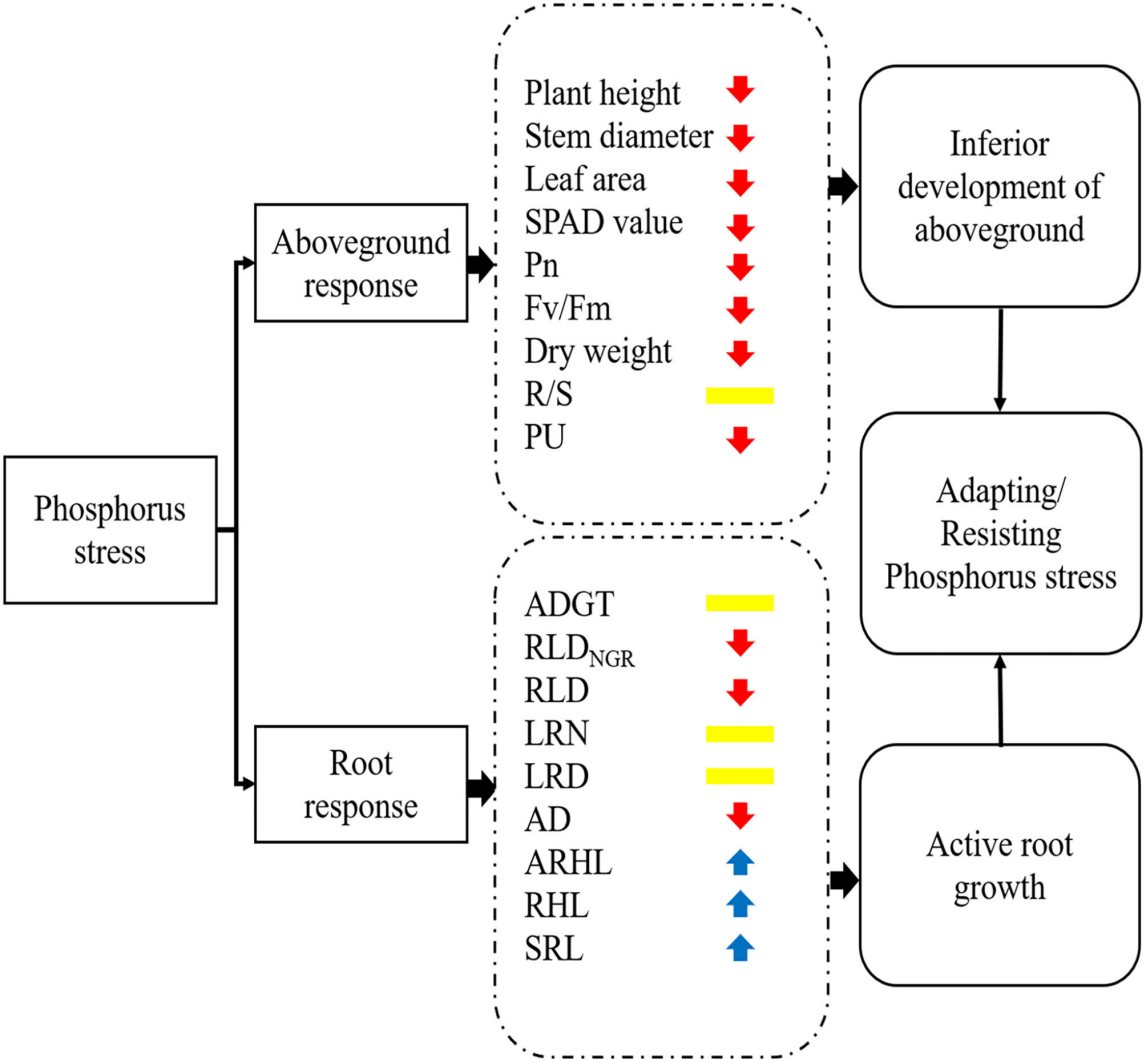
A working model of the response of cotton to phosphorus stress. The colors of arrows indicate that the morphological/physiological indicators were increased (blue) or decreased (red) or not significantly different (yellow) under phosphorus stress. Pn, net photosynthetic rate; *F*_v_/*F*_m_, maximum photochemical efficiency; R/S, root/shoot ratio; PU, phosphorus uptake; ADGT, average daily growth of taproots; RLD_NGR_, net growth rate per unit volume; RLD, root length density per unit volume; LRN, number of lateral roots; LRD, lateral root branching density; AD, average root diameter; ARHL, average root hair length; RHL, root hair life span; SRL, specific root length.

A simplified sketch of the response of cotton to P stress is provided in Figure 11. In this study, low P stress inhibited the growth and development of cotton seedlings and reduced the photosynthetic rate, chlorophyll content, and dry matter accumulation. In response to low P stress, cotton seedlings underwent significant modifications in their root system, including decreased AD and RLD; increased LRN, LRD, and SRL; and increased root hair length and longevity. These changes increased the surface area of the root and maximized the P absorption. The findings of this study show that RLD_NGR_, RLD, RDW, SRL, R/S ratio, PU efficiency, ARHL, and ADGT are important indicators of cotton root phenotypes under low P stress. Ultimately, this study suggests that optimizing root phenotype is an important way for plants to increase PU and P accumulation under PD conditions.

## Data Availability Statement

The original contributions presented in the study are included in the article/Supplementary Material, further inquiries can be directed to the corresponding authors.

## Author Contributions

ZZ, LL, and CL conceived the study and proposed the methods. LL, ZZ, LZ, and NW contributed to the preparation of equipment and acquisition of data. ZZ and LL wrote the code and tested the methods. HS, KZ, DL, YZ, AL, and ZB validated the results. ZZ wrote the manuscript. LL and CL revised the manuscript. All authors read and approved the final manuscript.

## Conflict of Interest

The authors declare that the research was conducted in the absence of any commercial or financial relationships that could be construed as a potential conflict of interest.

## Publisher's Note

All claims expressed in this article are solely those of the authors and do not necessarily represent those of their affiliated organizations, or those of the publisher, the editors and the reviewers. Any product that may be evaluated in this article, or claim that may be made by its manufacturer, is not guaranteed or endorsed by the publisher.

## Supplementary Material

Supplementary Figure 1 Image of cotton plant phenotypes under different phosphorus treatments. On the left side of **(A)** is “Nongdamian No. 10” cultivar under replete phosphorus conditions (ND-PR) and on the right side is “Nongdamian No. 10” cultivar under low phosphorus (ND-PD). On the left side of **(B)** is “Jimian 315” cultivar under replete phosphorus conditions (JM-PR) and on the right side is “Jimian 315” cultivar under low phosphorus (JM-PD).
