# Supplementary figures and images for: In situ Root Phenotypes of Cotton Seedlings Under Phosphorus Stress Revealed Through RhizoPot
Source: Front Plant Sci. 2021 Aug 30;12:716691. doi: 10.3389/fpls.2021.716691 (PMC8435733; doi:10.3389/fpls.2021.716691)

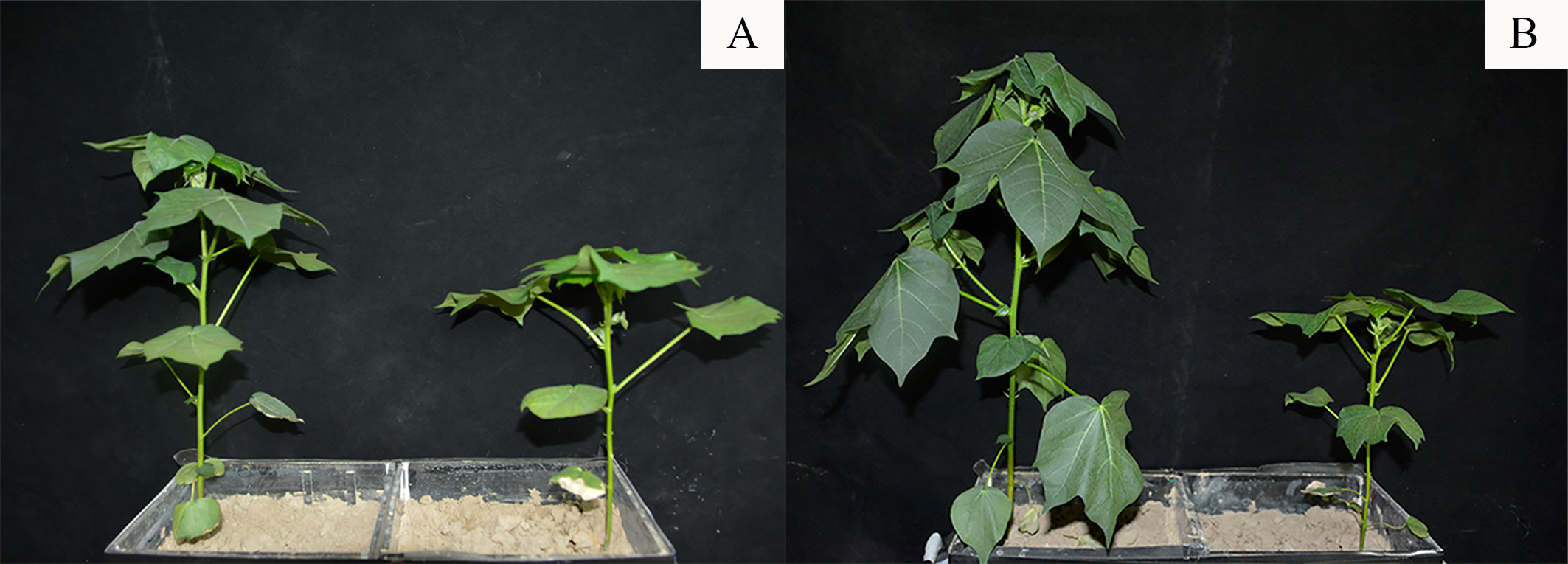

Supplement: Supplementary Figure 1 — Image of cotton plant phenotypes under different phosphorus treatments. On the left side of (A) is “Nongdamian No. 10” cultivar under replete phosphorus conditions (ND-PR) and on the right side is “Nongdamian No. 10” cultivar under low phosphorus (ND-PD). On the left side of (B) is “Jimian 315” cultivar under replete phosphorus conditions (JM-PR) and on the right side is “Jimian 315” cultivar under low phosphorus (JM-PD). [file Image_1.TIF]
